# Frequency of medically attended adverse events following tetanus and diphtheria toxoid vaccine in adolescents and young adults: a Vaccine Safety Datalink study

**DOI:** 10.1186/1471-2334-9-165

**Published:** 2009-10-05

**Authors:** Lisa A Jackson, Onchee Yu, Edward A Belongia, Simon J Hambidge, Jennifer Nelson, Roger Baxter, Allison Naleway, Charlene Gay, James Nordin, James Baggs, John Iskander

**Affiliations:** 1 Group Health Research Institute, Seattle WA, USA; 2 Epidemiology Research Center, Marshfield Clinic Research Foundation, Marshfield WI, USA; 3 Kaiser Permanente Institute for Health Research and Denver Health Community Health Services, Denver CO, USA; 4 Department of Biostatistics, University of Washington, Seattle WA, USA; 5 Kaiser Permanente Vaccine Study Center, Oakland CA, USA; 6 Kaiser Permanente Northwest, Portland OR, USA; 7 Department of Ambulatory Care and Prevention, Harvard Pilgrim Health Care and Harvard Medical School, Boston MA, USA; 8 HealthPartners, Minneapolis MN, USA; 9 Centers for Disease Control and Prevention, Atlanta GA USA

## Abstract

**Background:**

Local reactions are the most commonly reported adverse events following tetanus and diphtheria toxoid (Td) vaccine and the risk of local reactions may increase with number of prior Td vaccinations.

**Methods:**

To estimate the risk of medically attended local reactions following Td vaccination in adolescents and young adults we conducted a six-year retrospective cohort study assessing 436,828 Td vaccinations given to persons 9 through 25 years of age in the Vaccine Safety Datalink population from 1999 through 2004.

**Results:**

Overall, the estimated risk of a medically attended local reaction was 3.6 events per 10,000 Td vaccinations. The lowest risk (2.8 events per 10,000 vaccinations) was found in the 11 to 15 year old age group. In comparison with that group, the event risks were significantly higher in both the 9 to 10 and 21 to 25 year old age groups. The risk of a local reaction was significantly higher in persons who had received another tetanus and diphtheria toxoid containing vaccine (TDCV) in the previous five years (incidence rate ratio, 2.9; 95% confidence interval, 1.2 to 7.2). Twenty-eight percent of persons with a local reaction to Td vaccine were prescribed antibiotics.

**Conclusion:**

Medically attended local reactions were uncommon following Td vaccination. The risk of those reactions varied by age and by prior receipt of TDCVs. These findings provide a point of reference for future evaluations of the safety profile of newer vaccines containing tetanus or diphtheria toxoid.

## Background

Local reactions are the most commonly reported adverse events following administration of tetanus and diphtheria toxoid containing vaccines (TDCVs), and the risk of local reactions may increase with the number of prior vaccinations [1]. Local reactions, and particularly reactions that result in a medical visit, are therefore an outcome of interest in postlicensure assessments of the safety of TDCVs. Three vaccines containing tetanus or diphtheria toxoid are currently licensed for routine use in adolescents and young adults in the United States. Of those, tetanus and diphtheria toxoid and acellular pertussis (Tdap) vaccine and quadrivalent meningococcal conjugate vaccine (Menactra) (which contains meningococcal polysaccharide covalently linked to a diphtheria toxoid protein carrier), were introduced in 2005 and are now part of the routine adolescent immunization schedule. The older tetanus and diphtheria toxoid (Td) vaccine has been used for over 40 years.

Interpretation of the findings of postlicensure safety assessments of Tdap and Menactra vaccines would be aided by knowledge of the expected risk of medically attended local reactions following Td vaccine. To estimate the risk of medically attended local reactions following Td vaccination in adolescents and young adults we conducted a six-year retrospective cohort study assessing 436,828 Td vaccinations given to members of the Vaccine Safety Datalink (VSD) population in the era prior to the introduction of Tdap and Menactra vaccines.

## Methods

The study population included members of seven managed care organizations (MCOs) participating in the VSD project, a collaborative project between the CDC and eight MCOs established in 1991 to monitor and evaluate vaccine safety [2,3]. This project collects data on more than 8.8 million members annually including information on demographics, health plan enrollment, vaccinations, and medical encounters. The study cohort included MCO members who had a Td vaccination recorded at the MCO when they were 9 through 25 years of age and from January 1, 1999 through December 15, 2004. Participating MCOs included Group Health, Northern California Kaiser Permanente, Kaiser Permanente Northwest, Kaiser Permanente Colorado, Harvard Pilgrim Healthcare and Harvard Vanguard Medical Associates, Marshfield Clinic, and HealthPartners. At the time of this study, the first three of the listed MCOs were funded through the VSD to conduct assessments in children and adults and the remaining sites were funded only for child assessments. Therefore, for this study, all seven sites contributed information on vaccines given to persons 9 through 17 years of age, and three sites also contributed information on vaccines given to persons 18 through 25 years of age. Vaccinations were identified from computerized immunization records maintained by each MCO.

The primary study outcome was a chart-confirmed medically-attended local reaction, and a secondary outcome was a chart-confirmed medically-attended illness possibly indicative of a hypersensitivity response. An important distinction between these outcomes is that while local reactions are relatively easily attributable to a preceding vaccination, signs and symptoms such as hives or rash that are consistent with a hypersensitivity response are non-specific and in many cases may be attributable to exposures or etiologies other than vaccination.

Outcome events were presumptively identified by selected *International Classification of Diseases 9*^*th *^*Revision Clinical Modification *(ICD9-CM) codes assigned to inpatient and outpatient medical encounters within six days following the Td vaccination date (Table 1). The ICD9-CM codes used to identify presumptive events were selected based on expert opinion and information from previous studies. Since diagnosis codes assigned on the day of vaccination (day 0) often represent pre-existing conditions, and since most adverse events following vaccination are not expected to result in a medical visit on the same calendar day as vaccination, presumptive cases were not defined by a diagnosis code of cellulitis, limb swelling, pain in limb, allergy unspecified, lymphadenitis, or urticaria assigned on the day of vaccination. Presumptive cases defined by one of the above listed codes who had also had that code assigned within the 30 days prior to vaccination were also excluded. Since immediate hypersensitivity events could lead to a medical visit on the day of vaccination, diagnosis codes most consistent with an immediate hypersensitivity reaction, or which were specific indicators of adverse events, assigned on days 0 through 6 were used to presumptively identify events.

**Table 1:** Algorithm to define presumptive events by *International Classification of Diseases 9*^*th *^*Revision Clinical Modification *(ICD9-CM) codes associated with medical encounters within six days following vaccination.

ICD9-CM code(s)	Description	Presumptive events defined by a diagnosis code assigned on the day of vaccination (day 0) through day 6 or by a diagnosis code assigned on day 1 through day 6
682.3, 682.8, 682.9	Cellulitis	Day 1 through day 6
729.81	Limb swelling	Day 1 through day 6
729.5	Pain in limb	Day 1 through day 6
995.3	Allergy unspecified	Day 1 through day 6
708.x	Urticaria	Day 1 through day 6
289.3, 683, 785.6	Lymphadenitis	Day 1 through day 6
999.3	Infection following infusion or vaccination	Day 0 through day 6
995.0, 999.4	Anaphylaxis	Day 0 through day 6
999.5	Serum reaction	Day 0 through day 6
999.9	Complication of medical care	Day 0 through day 6
995.2	Adverse effect of a drug or biological substance	Day 0 through day 6

Presumptive cases defined by the above criteria were then validated by medical record review to confirm the occurrence of a medically-attended local reaction or an illness possibly indicative of a hypersensitivity response to Td vaccine and to collect additional information on the characteristics of those events. A validated medically-attended local reaction was defined by reported signs or symptoms consistent with a local reaction (inflammation, ulceration, erythema, warmth, swelling, pain, or tenderness) that were not known to have onset prior to vaccination and were not clearly due to another known cause (for example, trauma or insect sting). A validated illness possibly indicative of a hypersensitivity reaction was defined by a report of signs or symptoms consistent with urticaria, hives, wheezing, respiratory collapse, hypotension, angioedema, or anaphylaxis that were not known to have onset prior to vaccination. For validated illnesses possibly indicative of a hypersensitivity reaction, medical record review was also conducted to determine whether the event could be confirmed to have met the Brighton Collaboration case definition of anaphylaxis [4].

We identified 1,097 presumptive events following 436,828 Td vaccinations. Due to resource constrains, the maximum number of presumptive events selected for chart review at each of the participating MCOs was limited to 200. Two sites had more than 200 presumptive events identified and so for those sites a random sample of 200 presumptive events was selected for confirmatory review. The total number of presumptive events identified for confirmatory review across all sites was 765. Of those, chart review could not be completed for 52 events because the medical record lacked information on the post-vaccination encounter and so chart review was completed for 713 presumptive events.

The study was approved by the Group Health Institutional Review Board and, as appropriate, by institutional review boards at each of the participating sites.

### Statistical methods

Since about a third of the presumptive events were not reviewed, calculation of outcome rates based only on chart review verified events would underestimate the true event rate. To obtain statistically valid estimates of the true event rates that account for those missing data we used multiple imputation, based on Rubin's method [5]. This involved three basic steps. First, we imputed values for the outcome status of the presumptive events that were not reviewed. That is, the presumptive events that were not reviewed were assigned an imputed value of "validated" or "not validated". The likelihood of assignment of a validated status was based on the site-specific validation rate of the presumptive events that were reviewed. That is, if 50% of presumptive events at a given site that were reviewed were validated, then there was a 50% likelihood that each presumptive event not reviewed at that site would be assigned an imputed value of validated. We then "filled in the blanks" by using those site-specific likelihoods to assign each of the presumptive events that were not reviewed a validation status. This step was repeated four more times, to create five datasets with completed outcome validated status, either as determined by chart review or as assigned by the imputation method.

Second, we conducted analyses of each of those five datasets. Specifically, for each dataset, we calculated the risk of each validated event per 10,000 vaccinations and used Poisson regression to estimate the rate ratio comparing validated outcome risks between age and other subgroups of interest. Last, we obtained final estimates and confidence intervals based on the results of the five analyses using multiple imputation averaging techniques that account for the uncertainty introduced by the imputed data.

To estimate the contribution of individual diagnosis codes to the validated outcomes, we calculated the proportion of validated events that were assigned each of the codes and the proportion of presumptive events assigned each of the codes that were validated. The latter analysis was restricted to the presumptive events that were reviewed.

## Results

A total of 436,828 Td vaccinations administered to 430,568 persons meeting the study eligibility criteria were identified (Table 2). Following the 436,828 Td vaccinations, 1,097 presumptive medically attended events were identified, 765 of those presumptive events were selected for chart review, and reviews were completed for 713. Of the 713 presumptive events with completed reviews, 103 events met the definition of a medically attended local reaction to Td vaccine and another 26 met the definition of a validated illness possibly indicative of a hypersensitivity reaction. Using multiple imputation, we further estimated that among the 384 presumptive events not chart reviewed (and therefore with a missing validated outcome status) there were an additional 56 local reactions and 35 possible hypersensitivity reactions. This yielded a total of 159 estimated medically-attended local reactions and 61 illnesses possibly indicative of a hypersensitivity response in the final analyses.

**Table 2:** Estimated risk of a validated medically attended local reaction by age at Td vaccination.

Age group, years	Number Td vaccinations	Estimated^**1 **^number of medically attended local reactions	**Estimated event risk (95% CI) per 10,000 Td vaccinations**^**2**^
9-10	21,188	13	6.3 (3.4, 11.5)
11-15	310,803	88	2.8 (2.1, 3.7)
16-20	65,971	24	3.6 (2.3, 5.6)
21-25	38,866	34	8.7 (4.9, 15.7)
All	436,828	159	3.6 (2.8, 4.7)

CI = confidence interval

^1^The number of events estimated based on multiple imputation applied to account for incomplete chart reviews.

^2^Compared with the 11-15 year old age group, the risk was significantly higher in the 9-10 year old age group (p = 0.01) and the 21-25 year old age group (p = 0.005). The risk among 21-25 year olds was also significantly higher than among 16-20 year olds (p = 0.01).

### Medically attended local reactions

#### Risk of medically attended local reactions by age group and sex

The estimated risk of medically attended local reactions was 3.6 events per 10,000 Td vaccinations administered (Table 2). The lowest risk (2.8 events per 10,000 vaccinations) was found in the 11 to 15 year old age group. In comparison with that group, the event risks were significantly higher in both the 9 to 10 and 21 to 25 year old age groups. Compared to the 16 to 20 year old age group, the risk among 21 to 25 year-olds was also significantly higher. The risk of a medically attended local reaction did not vary significantly by sex [3.9 events per 10,000 Td vaccinations for females and 3.4 events per 10,000 Td vaccinations for males (p = 0.4)].

#### Association of prior vaccination with risk of medically attended local reactions

The risk of a medically attended local reaction to Td vaccine was compared between persons who did and who did not have a record of receipt of a TDCV within the previous five years. Prior vaccinations classified as TDCVs included Td as well as diphtheria, tetanus and pertussis vaccines administered as part of the childhood vaccination series but did not include Tdap or Menactra vaccines as those vaccines were not available during the time period of interest. In order to ensure accurate assessment of prior vaccinations from the MCO records, the analyses of TDCVs given before the Td vaccination were restricted to persons with at least five years of continuous enrollment in the MCO prior to the Td vaccination of interest. Overall and in the younger age groups there was a trend toward a higher risk of a local reaction in persons who received Td within five years of a prior TDCV but only the difference in all age groups combined was statistically significant (Table 3).

**Table 3:** Risk of a validated medically attended local reaction following Td vaccination, by history of receipt of tetanus and diphtheria toxoid containing vaccinations^1 ^in the prior five years, among persons with at least five years of enrollment in the MCO prior to the Td vaccination.

Age, years	History of receipt of TDCV in five years prior to Td vaccination	Number Td vaccinations	**Estimated number of local reactions**^**2**^	Event risk (95% CI) per 10,000 Td vaccinations	Incidence rate ratio (95% confidence interval) comparing those with and without a history of a TDCV in the prior five years
9-10	Yes	1,161	2	17.2 (2.9, 53.2)	4.6 (0.6, 35.4)
	No	8,015	3	3.7 (0.8, 17.2)	
11-15	Yes	2,876	2	8.2 (2.1, 32.6)	3.0 (0.8, 11.4)
	No	160,933	44	2.7 (1.8, 4.1)	
16-20	Yes	1,962	1	5.9 (0.8, 42.5)	1.8 (0.2, 15.6)
	No	27,713	9	3.3 (1.3, 8.2)	
21-25	Yes	165	0	0	Not estimable
	No	6,526	6	8.7 (3.1, 24.2)	
All	Yes	6,164	6	9.0 (3.7, 22.2)	2.9 (1.2, 7.2)
	No	203,187	63	3.1 (2.2, 4.4)	

CI = confidence interval

^1 ^Includes Td vaccine as well as diphtheria, tetanus and pertussis vaccines administered as part of the childhood vaccination series.

^2^Based on multiple imputation; the total of the age-group specific estimates may not add up to the overall total due to rounding average values across imputed datasets.

#### Characteristics of validated medically attended local reactions as defined by medical record review

Of the 103 chart review validated local reactions, 97 were reported to be associated with local edema, erythema, or swelling and for 59 of those the size of the involved area was noted. Of those, the distribution of the maximum reported diameter of the local reaction was <10 cm for 83%, 10 to <15 cm for 10%, and ≥ 15 cm for 7%. Of the four reactions reported to have a maximum diameter of ≥ 15 cm, three were reported to involve the entire upper arm and one of those extended to or past the elbow.

Of the 103 validated local reactions, 39 were noted to have been associated with pain, two were associated with ulcerated skin lesions, and 11 with lymphadenopathy. Fever was uncommon; only five persons were documented to have a temperature of 100°F or higher at the medical evaluation. No persons were evaluated in the hospital, 25 were evaluated in the ED or urgent care, and the remaining 78 were evaluated in outpatient clinics.

Twenty-three persons were given a clinical diagnosis of cellulitis. Of those, four persons were treated with parenteral antibiotics and 16 with oral antibiotics. Of the 80 persons with confirmed local reactions who were not assigned a diagnosis of cellulitis, two were treated with parenteral antibiotics and seven with oral antibiotics.

#### Diagnosis codes assigned to validated local reactions

The most common codes assigned to validated local reaction events included codes for cellulitis, serum reaction, complication of medical care, and adverse effect of a drug or biological substance (Additional file 1). Together these codes accounted for 83 of the 103 validated events. In general, however, the positive predictive value of diagnosis codes for validated local reactions was low, with the exceptions being the code for serum reaction, with a positive predictive value of 78%, and infection following vaccination, with a positive predictive value of 50%.

### Validated illness possibly indicative of a hypersensitivity reaction

The estimated risk of a validated illness possibly indicative of a hypersensitivity reaction, based on the 61 estimated events, was 1.4 events per 10,000 vaccinations. Of the 26 events that were confirmed by chart review to have met the study definition of an illness possibly indicative of a hypersensitivity reaction, eight had hives or urticaria without signs or symptoms involving other body systems, seven had non-urticarial rashes without signs or symptoms involving other body systems, seven had a clinical syndrome possibly consistent with an allergic reaction with signs and symptoms other than rash, and four had other clinical syndromes meeting the case definition. In many cases the clinical syndrome was attributed to another cause, such as exposure to an antibiotic or other medication or a concomitant vaccination.

Of the seven illnesses possibly consistent with an allergic reaction with signs and symptoms other than rash (with or without rash), in most cases there was limited information in the medical record and for all but one the available documentation did not provide information to confirm that the event met the Brighton Collaboration case definition of anaphylaxis. One case did meet that definition, with Level 1 of diagnostic certainty, but this was a 15 year old with known peanut allergy who had anaphylaxis after eating peanuts the day after Td vaccination.

## Discussion

In this study we found that validated medically attended local reactions were uncommon following Td vaccination, with an overall estimated risk of 3.6 events per 10,000 Td vaccinations. The risk of medically-attended local reactions varied by age, and was significantly higher in the 9 to 10 and in the 21 to 25 year old age groups compared with the risk in persons 11 to 20 years of age. In addition, among the subgroup of persons with at least five years of enrollment in the MCO prior to the Td vaccination, there was a statistically significant association between receipt of a TDCV within five years prior to the Td vaccination and a higher risk of a medically attended local reaction to Td vaccine.

The elevated risk in the youngest age group is consistent with the findings of Scheifele and colleagues, who reported greater injection site morbidity following Td vaccination in 11 to 12 year olds compared with 14 to 16 year olds [6]. This may have been due to the shorter interval between completion of the DTaP vaccination series and the Td vaccination in the younger age group. In our study, it is also possible that differences in health care seeking behavior among children and young adults contributed to the observed differences in risk of local reactions resulting in a medical visit. That is, if adolescents 11 through 15 years of age were less likely to seek medical care for a local reaction than both younger and older persons this may have contributed to the differences in risk of medically attended local reactions observed.

The Advisory Committee on Immunization Practices (ACIP) has recommended a 10-year minimum interval for routine administration of Td, although Td may be given for prophylaxis of wounds that are neither clean nor minor if there is no history of Td in the previous five years. For adolescents, ACIP has recommended a minimum interval of 5 years between the last pediatric DTaP and the adolescent Td dose [7,8]. Accordingly, in the VSD population, administration of Td within five years of a prior TDCV was uncommon, and was reported in only 3% of the subgroup of study subjects with at least five years of MCO enrollment. Given the rarity of our outcome of medically attended local reactions, we had a limited ability to identify an increased risk of these events among the relatively small subgroup of persons who had received a TDCV in the previous five years but our findings support the minimum intervals for Td vaccination recommended by ACIP. It should be noted, however, that those minimum intervals do not apply to administration of Tdap vaccine after Td vaccine. Data from clinical studies support the safety of administration of Tdap vaccine after Td vaccine at intervals of less than five years [9] and current recommendations indicate that Tdap may be given at shorter intervals in order to provide protection against pertussis [10,11].

Of the local reactions that were identified, for most the area of local edema, erythema, or swelling was relatively limited. Of the reactions noted to have local edema, erythema, or swelling and with a reaction size noted, for 83% the maximum diameter of edema, erythema or swelling was reported as less than 10 cm. Three local reactions were reported to involve the entire upper arm, and so may represent extensive limb swelling reactions, which have been reported in children following the fourth and fifth doses of DTaP vaccine [12-17], and which have been identified from Vaccine Adverse Event Reporting System (VAERS) reports in children and adults following Td, Tdap (unpublished VAERS data), and many other vaccines [18].

Persons with a medically attended local reaction were frequently given a clinical diagnosis of cellulitis and most of those given a diagnosis of cellulitis were prescribed antibiotic treatment. In addition, 11% of persons not given a diagnosis of cellulitis were also treated with antibiotics. Overall, 28% of persons with a medically-attended local reaction following Td vaccination were treated with antibiotics. This is similar to the proportion of children with medically attended local reactions following the 4^th ^or 5^th ^dose of DTaP who were prescribed antibiotics (21%) in a prior postlicensure study of DTaP vaccine conducted in the Group Health population [12]. Since infection is an unlikely explanation for injection site inflammation occurring within one or two days of Td vaccination, increasing provider awareness of the potential for significant injection site reactions following Td vaccine may decrease the potentially inappropriate use of antibiotics for those reactions. The Department of Defense Vaccine Centers Healthcare Network website http://www.vhc.info.org also provides algorithms for assessment and treatment of vaccine local reactions that may provide useful guidance for physicians who provide immunization services or evaluate vaccine adverse events.

In contrast to local reactions, the signs and symptoms occurring in persons meeting the definition of a validated illness possibly indicative of a hypersensitivity reaction could generally not be definitively attributed to vaccination. We also did not identify any case of anaphylaxis that could be attributed to Td vaccine in this study. This is consistent with a prior study in the VSD of children up to 17 years of age who were vaccinated during 1991 through 1997, which identified no cases of anaphylaxis following 152,636 Td vaccinations [19].

Two diphtheria toxoid containing vaccines, Tdap and Menactra, were introduced in 2005 and are included in the routine adolescent immunization schedule. Adolescents who receive these vaccines may be exposed to increased levels of diphtheria toxoid, which could be associated with an increased risk of local reactions. While it is possible that the safety profiles of Tdap and Menactra vaccines may differ from the safety profile of Td vaccine, the results of this study suggest that medically attended local reactions are likely to be uncommon following the more recently introduced diphtheria toxoid containing vaccines. These findings provide a point of reference for future evaluations of the safety profile of newer vaccines.

## Conclusion

Medically attended local reactions were uncommon following Td vaccination. The risk of those reactions varied by age and by prior receipt of TDCVs.

## Competing interests

RB has received research grants Sanofi Pasteur, the manufacturer of Td vaccine distributed in the United States. LAJ has received research grants from and served as a consultant to Sanofi Pasteur. The other authors declare that they have no competing interests.

## Authors' contributions

LAJ conceived of the study and drafted the initial protocol. All authors participated in the creation of the final protocol. LAJ, EAB, SJH, RB, AN, and CG provided oversite for the data collection at their study site. OY and JN drafted the study analysis plan and performed the study analyses. LAJ drafted the manuscript and all authors read and approved the final manuscript.

## Pre-publication history

The pre-publication history for this paper can be accessed here:

http://www.biomedcentral.com/1471-2334/9/165/prepub

## Supplementary Material

Additional file 1 **Sensitivity and positive predictive value (PPV) of individual ICD9 codes for identification of validated local reactions and validated events possibly indicative of a hypersensitivity response to Td vaccination**. The data provided report the proportion of validated events that were associated with specific ICD9 codes. Click here for file
