# Supplementary material for: Frequency of medically attended adverse events following tetanus and diphtheria toxoid vaccine in adolescents and young adults: a Vaccine Safety Datalink study
Source: BMC Infect Dis. 2009 Oct 5;9:165. doi: 10.1186/1471-2334-9-165 (PMC2765445; doi:10.1186/1471-2334-9-165)
Supplement: Additional file 1 — Sensitivity and positive predictive value (PPV) of individual ICD9 codes for identification of validated local reactions and validated events possibly indicative of a hypersensitivity response to Td vaccination. The data provided report the proportion of validated events that were associated with specific ICD9 codes. [file 1471-2334-9-165-S1.DOC]

Table 4. Sensitivity and positive predictive value (PPV) of individual ICD9 codes for identification of validated local reactions and validated events possibly indicative of a hypersensitivity response to Td vaccination.

| ICD9 code(s) | Description of ICD9 code | No. presumptive cases (i.e., with any identifying code) (N=1097) | No. selected for chart review (N=765) | No. with completed chart review (N=713) | No. of chart review validated local reactions with code* (n=103) | % of validated local reactions with code | % of events with the code that were validated local reactions | No. of chart review validated HS reactions (n=26) | % of validated HS reactions with code | % of events with the code that were validated HS reactions |
| --- | --- | --- | --- | --- | --- | --- | --- | --- | --- | --- |
| 682.3, 682.8, 682.9 | Cellulitis | 410 | 235 | 218 | 22 | 21 | 10 | 1 | 4 | 0.5 |
| 729.81 | Limb swelling | 15 | 13 | 9 | 2 | 2 | 22 | 1 | 4 | 11 |
| 729.5 | Pain in limb | 93 | 67 | 59 | 7 | 7 | 12 | 0 | 0 | 0 |
| 995.3 | Allergy unspecified | 92 | 44 | 41 | 8 | 8 | 19 | 16 | 61 | 39 |
| 708.x | Urticaria | 0 | 0 | 0 | 0 | 0 | - | 0 | 0 | 0 |
| 289.3, 683, 785.6 | Lymphadenitis | 85 | 60 | 57 | 8 | 8 | 14 | 2 | 8 | 3 |
| 999.3 | Infection following infusion or vaccination | 4 | 4 | 4 | 2 | 2 | 50 | 0 | 0 | 0 |
| 995.0, 999.4 | Anaphylaxis | 10 | 10 | 10 | 0 | 0 | 0 | 0 | 0 | 0 |
| 999.5 | Serum reaction | 25 | 24 | 23 | 18 | 17 | 78 | 3 | 11 | 13 |
| 999.9 | Complication of medical care | 73 | 63 | 61 | 18 | 17 | 29 | 0 | 0 | 0 |
| 995.2 | Adverse effect of a drug or biological substance | 314 | 261 | 243 | 25 | 24 | 10 | 3 | 11 | 1 |

HS=hypersensitivity

*Five confirmed local reactions had two, and one had three, of the ICD9 codes used to identify presumptive events assigned. The codes assigned for the six events were swelling, pain and serum reaction; lymphadenitis and complications of medical care; pain in limb and allergy; cellulitis and serum reaction; cellulitis and allergy; and cellulitis and limb swelling.
